# Time trends and persistence of the return difference between growth and value investment strategies

**DOI:** 10.1371/journal.pone.0332690

**Published:** 2025-09-23

**Authors:** Manuel Monge, Rafael Hurtado, Juan Infante

**Affiliations:** 1 Universidad Francisco de Vitoria, Madrid, Spain; 2 Universidad Europea de Madrid, Madrid, Spain; 3 CUNEF Universidad, Madrid, Spain; 4 Universidad Villanueva, Madrid, Spain; Alexandru Ioan Cuza University: Universitatea Alexandru Ioan Cuza din Iasi, ROMANIA

## Abstract

This paper examines the dynamic disequilibrium between value investing and growth strategies, focusing on the structural changes induced by the COVID-19 pandemic. Using fractional integration and Markov-switching dynamic regression (MS-DR) models, we analyze persistence and regime shifts. The results reveal that, prior to March 2020, the return difference was in a regime of high persistence and no reversion to the mean, making the deviations long-lasting. After the pandemic, the system shifted to a regime of moderate persistence with reversion to the mean, indicating that the return differences now tend to correct over time. This regime shift, confirmed by the Markov switching model, highlights a permanent change in the dynamics of value and growth strategies, which significantly affects their long-term equilibrium.

## 1. Introduction

In the field of finance, representing two fundamentally different approaches to identifying and capitalizing on opportunities in financial markets, value and growth investment strategies have long been a subject of academic and practical debate. Both strategies are based on differing beliefs and preferences regarding growth expectations, long-term versus short-term profitability, and the choice between more stable or volatile assets [1,2].

The value investing strategy focuses on identifying stocks that are undervalued by the market based on fundamental criteria, with the expectation that their price will correct over time. This approach was introduced by Graham et al. [3] after conducting a thorough analysis supported by both qualitative and quantitative arguments. Value investors seek stocks whose current market price is below their intrinsic value, trusting that the market will eventually correct this discrepancy. Lakonishok et al. [4] and other studies have shown that value investments tend to offer higher returns than growth strategies during periods of economic stability, though they may underperform during recessions.

On the other hand, the growth investing strategy prioritizes companies with above-average market growth expectations, anticipating that their future expansion will generate significant returns. These companies often show superior earnings growth, with the expectation that they will continue to deliver high values in the future [5]. Various studies have explored growth strategies, such as venture capital investments [6,7] and business partnerships [8], which tend to focus on innovative companies with high expansion potential. However, despite the promise of high returns, these investments also involve greater volatility and risk.

The extant literature has thoroughly examined the value vs. growth debate across various markets and time periods. For example, Capaul et al. [9] studied the performance of both value and growth investments in France, Germany, Switzerland, England, Japan, and the United States through the price/earnings ratio, finding that value stocks outperformed growth stocks in all markets during the period analyzed. Similarly, Rosenberg et al. [10] and Fama and French [11] argued that the outperformance of value stocks may be linked to time-varying risk. They suggested that the risk associated with non-value growth strategies is higher during economic downturns when the risk premium is also high, and lower during economic booms when the risk premium declines.

Building on this, Petkova and Zhang [12] examined the risk associated with value and growth stocks, finding that the time-varying risk can explain the value premium. Their analysis revealed that value betas tend to covary positively with expected market risk, while growth betas covary negatively. Additionally, Reuer and Tong [13] pointed out that the outperformance of value investing remains controversial. While some researchers attribute it to the higher risk associated with value stocks, others argue that the rewards come from cognitive biases underlying investor behavior, as highlighted by Barberis and Thaler [14].

Moreover, when conditional volatilities are high, the expected excess returns of value stocks tend to be more sensitive to economic conditions than those of growth stocks. This suggests that the expected value premium varies over time, spiking during periods of high volatility and gradually decreasing thereafter [15]. Cronqvist et al. [16] further suggested that investors who have experienced adverse macroeconomic conditions may be more reluctant to invest in growth stocks, turning instead to value investments. Guidolin and Timmermann [17] showed that return premia, volatilities, and correlations between size and value portfolios vary substantially across latent economic regimes, suggesting that regime shifts play a critical role in shaping asset pricing anomalies and optimal asset allocation. Sandy Nairn from the Templeton Global Equity Group underscored the relative insignificance of methodological discussions regarding indices, echoing the sentiment of Fama [18].

The dilemma between value and growth intensifies when their long-term performance is analyzed. While growth companies may outperform value stocks at certain times, as shown by Beneda [19] for portfolios constructed between 1990 and 2000, studies also indicate that, after a few years, growth stocks tend to underperform relative to value stocks. This suggests that the success of one strategy over the other largely depends on market conditions and the investment time horizon.

The importance of understanding these strategies lies in their ability to offer complementary approaches to investors. While value investing has proven resilient through various economic cycles, as suggested by Asness et al. [20], growth investing has captured emerging opportunities in dynamic sectors, albeit with higher risk. The evaluation of these strategies has expanded through modern techniques such as unit root models and stationarity tests [21,22], which have allowed for the analysis of the relationship between projected and actual earnings in both approaches.

The COVID-19 pandemic introduced unprecedented levels of economic uncertainty and disruption, prompting investors to reassess traditional investment paradigms. The pandemic’s impact on global markets has been particularly notable in the context of value versus growth investing. Prior to the pandemic, growth stocks—particularly those in the technology sector—outperformed value stocks significantly. However, as the global economy shifted in response to the pandemic, questions arose about the long-term viability of these trends and whether the performance gap between value and growth stocks would persist or reverse in the post-pandemic era [23,24].

The line of research initiated by Monge et al. [25] concluded that growth and value-based investment strategies experienced a significant trend change after the COVID-19 pandemic. Building on the results and conclusions of their research, this scientific paper aims to further explore the dynamic behavior of both investment strategies by analyzing their return difference over an extended period, from January 2009 to October 2024.

We analyze the return difference between value and growth strategies using several advanced methodologies. First, we employ fractional integration to examine the long memory properties of the return difference between the two investment strategies. This approach allows us to determine whether the disequilibrium is driven by long-run deviations that slowly revert to equilibrium or whether shocks have permanent effects. Additionally, it helps us assess whether the disequilibrium exhibits strong trend components or tends to revert to a stable level over time, thus detecting non-stationarity and long-run dependencies in the investment dynamics.

Furthermore, we utilize the Markov-switching dynamic regression (MS-DR) model to identify whether the dynamics of the disequilibrium differ across regimes; one regime may represent a period of high persistence and no reversion, while another indicates moderate persistence with a tendency toward mean reversion. This model also allows us to capture probabilistic transitions between these regimes, reflecting how market conditions or external shocks lead to changes in the behavior of the disequilibrium over time. This approach provides a comprehensive understanding of the varying dynamics and regime shifts that impact the return difference between value and growth investment strategies.

The structure of this paper is as follows. Section 2 describes the data used for our study. Section 3 explains the methodologies used to carry out the research. The results are discussed in Section 4 and, finally, the conclusions are found in Section 5.

## 2. Data

The data used in this study to analyze the behavior of the return difference between value and growth investment strategies were obtained from the Thomson Reuters Eikon platform and consist of the following two indices:

- MSCI International World Index Value Price Index USD Real-time
- MSCI International World Index Growth Price Index USD Real-time

We use the original MSCI index levels rather than rebased (e.g., base-100) versions. This preserves the integrity of return differentials and structural patterns, as our econometric methods are invariant to such linear transformations.

These indices were retrieved at daily frequency for the period from January 23, 2009 to October 7, 2024. The return difference series analyzed in this study was constructed by taking the daily arithmetic difference between the level values of the Value and Growth indices:


$${Return\;differences}_{t}={Value}_{t}-{Growth}_{t}$$

**Fig 1:**
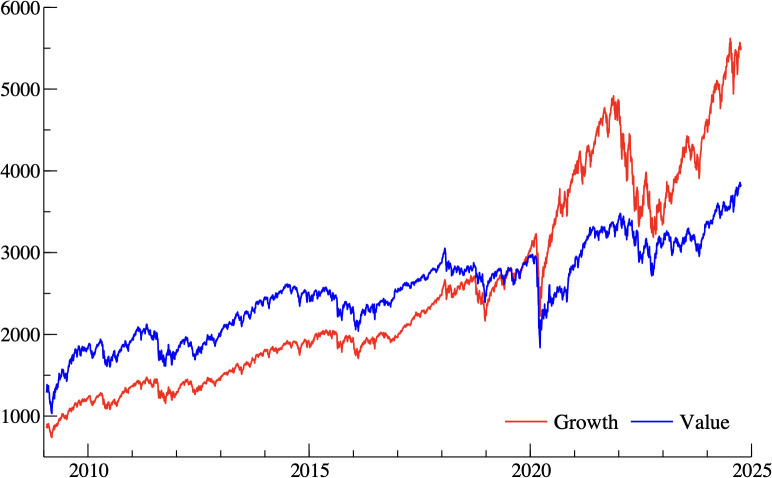
Performance of growth (red line) and value (blue line) equity investment strategies from 2009 to 2025. Notes: The figure plots the daily levels of the MSCI World Growth Index (red line) and MSCI World Value Index (blue line) between January 2009 and October 2024. The vertical axis shows index levels in USD, and the horizontal axis represents time.

Fig 1 illustrates the comparative performance of the value and growth investment strategies over the entire sample period.

**Fig 2:**
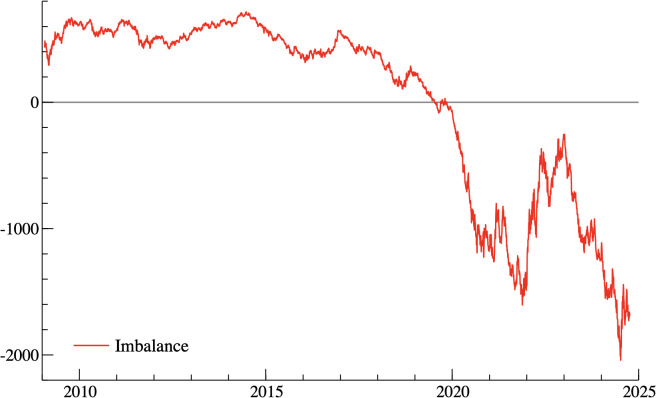
Return differential between growth and value equity investment strategies from 2009 to 2025. Notes: The figure displays the daily arithmetic difference between the MSCI World Value and MSCI World Growth indices (Value – Growth). Negative values indicate growth outperforming value, while positive values indicate value outperforming growth. The vertical axis measures the return spread, and the horizontal axis shows time.

Fig 2 shows the evolution of the return difference between value and growth investment strategies during the same period mentioned above. Between January 2009 and March 2020, the return difference fluctuates around a small range, generally staying close to zero, indicating that the differences between the performance of value and growth strategies were relatively minor. Starting in March 2020, coinciding with the onset of the COVID-19 pandemic, there is a sharp and significant increase in the return difference, with values consistently dropping below zero. This indicates that growth stocks likely outperformed value stocks by a large margin, creating a growing divergence between the two strategies. The pandemic appears to have acted as a shock, leading to a prolonged period where growth outpaced value.

## 3. Methodology

a. **ARFIMA (p, d, q) model**

Thanks to authors such as Diebold and Rudebusch [26], Hassler and Wolters [27], Lee and Schmidt [28], and others, it is now widely accepted that all unit root algorithms have incredibly low power if the real data creating process is fractionally integrated or has long memory. Fractional orders of differentiation are thus permitted in the following.

Because of this, we employ the ARFIMA (p, d, q) model, using the following mathematical notation:


$${\left(\text{1}-L\right)}^{d}x_{t}=u_{t},\;t=\text{1},\;\text{2},$$ (1)

According to Equation (1), $u_{t}$ denotes I(0), L is the lag-operator $\left(Lx_{t}=x_{t-\text{1}}\right)$, d is any real number, and $x_{t}$ is the time series with an integrated process of order d $\left(x_{t}\approx I(d)\right)$.

The advantages of using the ARFIMA (p, d, q) model over any unit root tests are several: 1) They allow fractional values for d providing greater flexibility in how the series is modeled; 2) They capture long-term dependence; 3) They offer a complete framework for modeling and predicting time series.

The Bayesian information criterion [29] and the Akaike information criteria [30] were used to determine the models’ correct AR and MA ordering.

For the time series and the subsamples, the d parameter has been estimated for each possible combination of AR and MA terms (p; q < 2), accounting for their 95% confidence bands.

b. **Markov Switching Dynamic Regression (MS-DR) model**

Our approach is based on the single-index Markov switching dynamic factor model, which combines comovements and business-cycle shifts into a statistical model, and was first developed in the mid-1990s by Kim and Yoo [31], Chauvet [32], and Kim and Nelson [33]. According to the model, a vector of N economic indicators, $\begin{array}{c} y_{t}=(y_{\text{1},t},\ldots ,\;y_{N,t}{)}^{\prime } \end{array}$, which are assumed to fluctuate in tandem with the state of the economy generally, can be broken down into the total of two parts. The common comovements are explained by the first component, $\begin{array}{c} f_{t}=(f_{\text{1},t},\ldots ,f_{r,t}{)}^{\prime } \end{array}$, which is a linear combination of r unseen factors.

The N×1 time series vector $u_{t}$, which depicts the peculiar movements in the series, is the second element. This implies the following formulation:


$$y_{t}=\Lambda f_{t}+u_{t}$$ (2)

where $u_{t}$ is the vector of idiosyncratic components and Λ is the N×r factor loading matrix.

We explain the business cycle asymmetries by supposing that an unobserved regime-switching state variable, $s_{t}$, controls the dynamic behavior of the common factors. The expansion and recession states at time t can be denoted by the labels $s_{t}=\text{0}$ amd $s_{t}=\text{1}$, respectively, in this paradigm. Furthermore, the conventional wisdom holds the state variable changes in accordance with an irreducible two-state Markov chain, the transition probabilities of which are determined by


$$p\left(s_{t}=j|s_{t-\text{1}}=i,\;s_{t-\text{2}}=h,\ldots ,I_{t-\text{1}}\right)=p\left(s_{t}=j|s_{t-\text{1}}=i\right)=p_{ij}$$ (3)

where i, j=0, 1, and $\left\{y_{\text{1}},\ldots ,\;y_{t}\right\}$ represents the information set up to period t. It is assumed that the common factor vector is subject to an autoregressive process with a switching intercept


$$f_{t}=\mu_{s_{t}}+\phi_{\text{1}}f_{t-\text{1}}+\ldots +\phi_{p}f_{t-p}+a_{t},$$ (4)

where $a_{t}$ is an independent variable of $s_{t}$ white noise with variance $\Sigma_{a}$. The basic idea that the comovements of the various time series originate from the common component is expressed by the primary identifying assumption in the model. By presuming that $u_{t}$ and $f_{t}$ are mutually uncorrelated at all leads and lags, this is accomplished (This theoretical section does not address additional identifying restrictions that are needed to estimate the model.).

The idiosyncratic error $\begin{array}{c} u_{t}=(u_{\text{1},t},\ldots ,u_{N,t}{)}^{\prime } \end{array}$ is assumed to have an autoregressive process of order $p_{i}$ for each element $u_{i,t}$.


$$u_{i,t}=\psi_{i,\text{1}}u_{i,t-\text{1}}+\ldots +\psi_{i,p_{i}}u_{i,t-p_{i}}+\epsilon_{i,t}$$

where a white noise process with variance $\sigma_{i,\;s_{t}}^{\text{2}}$ is represented by $\left\{\epsilon_{i,t}\right\}$. The idiosyncratic component’s dynamics are expressed in matrix form as


$$u_{t}=\psi_{\text{1}}u_{t}+\ldots +\psi_{p}u_{t-P}+\epsilon_{t}$$

where $\Psi_{j}=diag(\psi_{\text{1},j},\ldots ,\psi_{N,j})$, $P=\text{max}(p_{\text{1}},\ldots ,p_{N})$, and $var\left(\epsilon_{t}\right)=diag(\sigma_{{\text{1},s}_{t}}^{\text{2}},\ldots ,\;\sigma_{{N,s}_{t}}^{\text{2}})$ (Finding the common factors in small-scale factor models requires assuming that the idiosyncratic components are uncorrelated in cross-section [34]. It is assumed that the idiosyncratic mistakes in large-scale models (N → ∞) have a weak correlation.).

## 4. Empirical results

The unit root/stationary test is the first study we perform in this research article. After determining that the return difference series exhibits non-stationary I(1) behavior using Augmented Dickey-Fuller (ADF) unit root [35], and due to the lower power of the unit root methods under fractional alternatives, we used more advanced methodologies based on fractional integration to not only confirm more accurately the non-stationarity of the data, but also the persistence of the data.

Table 1 displays the estimates of the fractional differencing parameter d and the AR and MA terms, using Sowell’s [36] maximum likelihood estimator of various ARFIMA (p, d, q) specifications with all combinations of p, q ≤2, for each time series.

**Table 1:** Long memory results using the original time series.

Data analyzed	Sample size (days)	Model Selected	d	Std. Error	Interval	I(d)
Original Data						
Return difference	4095	ARFIMA (1, d, 2)	1.04	0.040	[0.98, 1.11]	I(I)
pre-COVID						
Return difference	2874	ARFIMA (2, d, 2)	1.05	0.072	[0.93, 1.17]	I(1)
post-COVID						
Return difference	1221	ARFIMA (2, d, 2)	0.99	0.024	[0.95, 1.03]	I(1)

Notes: This table reports the estimates of the fractional differencing parameter (d) and autoregressive/moving average terms of ARFIMA (p,d,q) models applied to the return differential between value and growth indices. Estimates are obtained using Sowell’s [36] maximum likelihood method. I(d) denotes the inferred order of integration. “Pre-Covid” corresponds to January 2009–March 2020; “Post-Covid” corresponds to March 2020–October 2024. Standard errors and 95% confidence intervals are reported. Abbreviations: AIC = Akaike Information Criterion; BIC = Bayesian Information Criterion.

We observe in Table 1 that the estimates of what we obtain by focusing on the original time series of the return differences between value and growth investment strategies are greater than 0.5 (d=0.5). According to this result, we can determine that since the result of the parameter d is 1.04, the unit root null hypothesis cannot be rejected and d is statistically significantly above 1. This result indicates that return differences exhibit long memory (high degree of persistence) and are non-stationary. In other words, the return difference has a strong trend component, and any shocks or changes can have a lasting impact, making it difficult for the series to revert to a previous trend or level without significant intervention. The high d value suggests that return differences are heavily influenced by historical trends and shocks, making them more prone to prolonged periods of deviations from any long-term equilibrium. This implies greater difficulty in managing or predicting future movements, as past shocks continue to influence the series for a long time.

Analyzing the time series by subsamples, we observe that, prior to the break, the time series with a d=1.05 follows the same behavior as the one we have discussed for the original series.

However, after the break, we observe that the subsample presents a priori a different behavior. As the parameter d is less than 1 (d=0.99), this implies that the subsample exhibits long memory, remaining non-stationary I(1) behavior; however, in the long term the series has some tendency to revert to its mean, albeit slowly.

This behavior that we are commenting on for the subsample after the break cannot be confirmed statistically. According to the confidence interval, the null hypothesis I(1) cannot be rejected, and, therefore, after the shock we would have a behavior of no reversion to the mean, as in the other two cases analyzed.

Since the results obtained before and after the break located in the time series in March 2020 present a change of regime, according to the change of behavior between the two subsamples we should use a Markov dynamic regression model based on two regimes to try to show and capture the change of dynamic structure before and after the break.

Table 2 shows the estimates of the Markov switching dynamic regression model using the maximum likelihood method.

**Table 2:** Estimation of the MS (2) dynamic regression model.

1) Descriptive statistics for scaled residuals					
**Normality test:**	$\chi^{\text{2}}=\text{1}\text{3}\text{6}.\text{7}\text{3}\;(\text{0}.\text{0}\text{0}\text{0}\text{0}{)}^{**}$				
**ARCH 1–1 test**	F(1,4089)=0.000024395 (0.0000)**				
**Portmanteau (63)**	$\chi^{\text{2}}(\text{6}\text{3})=\text{0}.\text{0}\text{0}\text{0}\text{0}\text{1}\text{7}\text{1}\text{6}\text{3}\;(\text{0}.\text{0}\text{0}\text{0}\text{0}{)}^{**}$				
**Linearity LR–test**	$\chi^{\text{2}}(\text{2})=\text{8}\text{0}\text{8}\text{1}.\text{3}\;(\text{0}.\text{0}\text{0}\text{0}\text{0}{)}^{**}$ aprox. upperbound: ${\left(\text{0}.\text{0}\text{0}\text{0}\right)}^{**}$				
**2) Estimation results from MS (2) for the water sector**					
	**Coefficient**	**Std. Error**	**t-value**	**t-prob**	
**Constant (0)**	444.786	4.989	89.2	0.000	
**Constant (1)**	–1019.51	7.839	–130	0.000	
**Sigma**	267.142	2.956			
**3) Regime classification based on smoothed probabilities**					
	**Start date**	**End date**	**Days**	**Avg. Prob.**	**Total**
**Regime 0**	2009-01-23	2020-03-03	2896	1.000	2896 days (70.72%) with average duration of 2896 days
**Regime 1**	2020-03-04	2024-10-07	1199	0.999	1199 days (29.28%) with average duration of 1199 days
**4) Transition probabilities (persistence of the regime)**					
		**Regime** 0, t	**Regime 1** , t		
	**Regime 0** , t+1	0.99966	0.0000		
	**Regime 1** , t+1	0.00033925	1.0000		

Notes: This table presents parameter estimates from the two-regime Markov Switching Dynamic Regression (MS-DR) model applied to the return differential. Regime 0 corresponds to high persistence with no mean reversion, while Regime 1 corresponds to moderate persistence with a tendency to mean reversion. Transition probabilities measure the likelihood of remaining in or switching between regimes. Sigma denotes residual volatility. Significance levels: p < 0.05 (*), p < 0.01 (**).

The adjusted model refers to the MS (2), which means that the model assumes two possible regimes: i=0 (high persistence and non-mean reversion) or i=1 (moderate persistence with a tendency to mean reversion). In section 1 of Table 2 we present the descriptive statistics for residuals, and provide important insights into the model’s behavior. In the normality test, the value obtained is significant at 5% and 1%, respectively. This result indicates that the data do not follow a normal distribution. The non-normality of residuals, confirmed by the Jarque-Bera test, suggests caution when interpreting p-values under the assumption of normality. However, the large sample size reduces the risk of biased inference, as the central limit theorem still provides valid approximations. On the other hand, according to the ARCH 1−1 test, it is observed that the variance of the errors is not constant over time and that its behavior depends on past observations. This effect indicates time-varying volatility in the residuals, a feature often observed in financial time series. This finding strengthens the case for regime-switching models, as shifts in volatility are often linked to structural changes in financial conditions.

For the Ljung-Box or Portmanteau test (63), the null hypothesis is again rejected in favor of the alternative. This means that there is autocorrelation (the series is not random) in at least one of the first 63 lags. This result suggests memory effects and intertemporal dependencies not fully captured by linear models.

Finally, the LR–test for linearity indicates that the result is again significant and suggests that a nonlinear model provides a better representation of the data. This further justifies the use of a nonlinear, dynamic Markov switching framework to model such features accurately.

In section 2 of Table 2 we present the results from the Markov switching model that we have estimated. It is noted that each of the regime-specific and intercepts obtained from the time series (constant for each regime) serves as an indicator to determine the average growth rate of return difference for a specific regime. The results indicate two markedly distinct regimes. As we can see in the states of high persistence and non-reversion to the mean, we obtain a result of 444.786, which indicates a very significant average growth of the return difference in this regime. This value reflects a positive growth of the return difference and how much of it remains uncorrected. For the state of moderate persistence with a tendency to mean reversion the value is −1019.51. This regime indicates that, during these periods, the average growth of the return difference is negative, suggesting a tendency to correct the return differences toward equilibrium.

Finally, the value of sigma is 267.142, which shows that there is considerable variation in growth rates within these regimes, indicating that movements in return differences are not perfectly uniform in each regime, but the overall behaviors are clearly differentiated.

**Fig 3:**
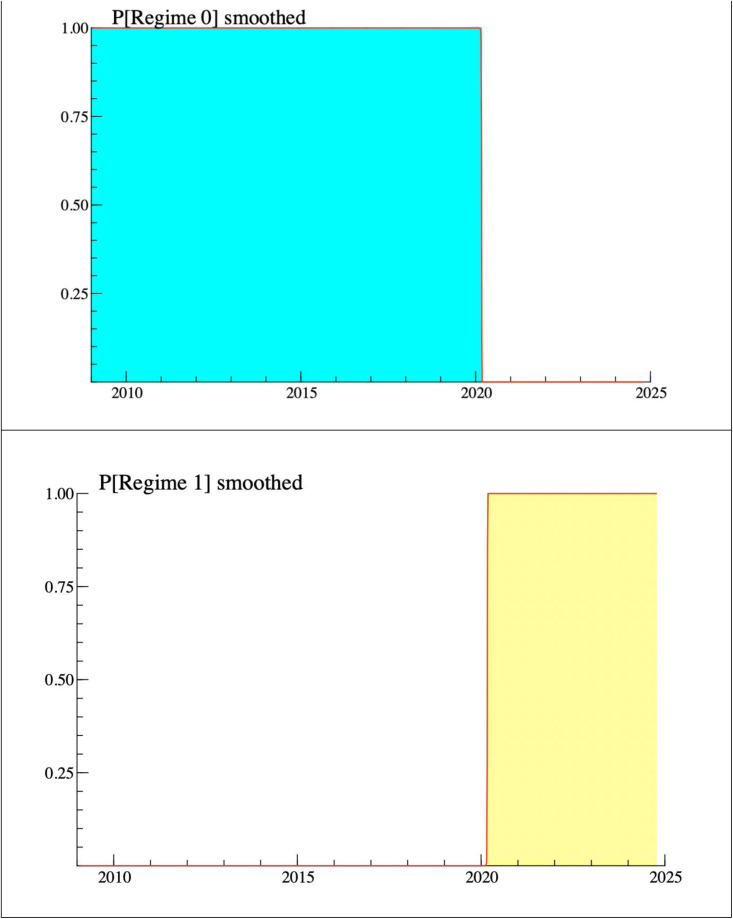
Graphical representation of the regimes. Notes: The figure illustrates the smoothed probabilities of being in each regime as estimated by the two-regime Markov Switching Dynamic Regression (MS-DR) model. Regime 0 (blue) corresponds to high persistence with no mean reversion, and Regime 1 (red) corresponds to moderate persistence with a tendency to mean reversion. The sample covers January 2009–October 2024.

Section 3 of the table places the focus on the durations of each regime for the time series we are analyzing. To explain this section, we wanted to make it more visual in order to make it understandable. For this reason, we have decided to represent the results obtained in this section in Fig 3.

According to Fig 3, which represents the estimated probabilities, the situation of high persistence and no reversion to the mean (Regime 0) is 1 (100%) from January 2009 to March 2020. This means that for 2896 days the series was practically constantly in this regime and the probability of being in Regime 1 during this period was zero.

From March 2020 we see a regime change (Regime 1), which corresponds to moderate persistence with a tendency to mean reversion, with an associated probability of 0.999 (99.9%). This means that for 1199 days the series was practically in this regime all the time. This suggests a structural change in the series, from a behavior of high persistence and no reversion to one in which the return differences begin to correct slowly, with a greater tendency toward mean reversion.

Finally, we focus on the transition probabilities in section 4 of Table 2, which reinforces the interpretation we discussed previously.

At first, we see that if the system is in Regime 0 at time t, there is a 99.966% probability that it will still be in Regime 0 in the next period (t+1). The transition probability from Regime 0 to Regime 1 is extremely low (0.0339%), suggesting that the system is unlikely to exit Regime 0, but is possible with rare events or significant shocks.

Once the system enters Regime 1, the probability of remaining in this regime is 100%. This indicates that Regime 1 is absorbing, i.e., once the system switches to this regime, it remains there with no chance of returning to Regime 0.

The Markov switching dynamic regression model used in this study assumes that each regime is governed by a separate set of parameters, with transitions between regimes determined endogenously through a first-order Markov process. The assumption of stationarity applies within each regime, meaning the dynamics are stable conditional on the regime.

The finding that Regime 1 (post-March 2020) is persistent, with a transition probability of 1.000, which is not imposed but emerges from the data. This may be interpreted as an absorbing regime, though future observations could alter this classification.

Additional model specifications with more than two regimes were also tested during the model selection process. However, these yielded either unstable parameters or higher AIC/BIC values, suggesting that the two-regime structure best captures the structural break and persistence behavior observed in the data.

These findings indicate that the regime shift observed after March 2020 is not only statistical but also consistent with structural changes in investor behavior documented in the literature. Several mechanisms may explain why return differences exhibit stronger mean reversion in Regime 1. First, the unprecedented monetary and fiscal interventions reduced systemic uncertainty and encouraged faster correction of mispricings [37,38]. Second, the unprecedented scope of mandatory shutdowns and voluntary social distancing not only curtailed economic activity, as emphasized by Baker et al. [39], but also reshaped investor risk preferences by heightening aversion to highly uncertain cash flows. This surge in perceived risk amplified flight-to-quality dynamics, thereby increasing the relative appeal of safer, value-oriented assets [40,41]. Such a shift in preferences is consistent with our empirical finding that, in the post-Covid regime, return differentials exhibit a greater tendency to revert toward equilibrium. Third, the accelerated adoption of digital platforms and the central role of algorithmic traders in modern equity markets helped sustain liquidity and price efficiency during the COVID-19 turmoil. Consistent with Chakrabarty and Pascual [42], stocks with higher algorithmic trading activity experienced a smaller deterioration in liquidity, greater competition in liquidity provision, and no significant loss of price efficiency compared to those with lower activity. This evidence suggests that algorithmic market making mitigated the persistence of disequilibria and contributed to stabilizing market functioning in the post-Covid regime. Taken together, these structural shifts suggest that the Covid-19 crisis permanently reshaped the dynamics of value and growth strategies, leading to a regime in which deviations tend to be corrected more rapidly and persist over shorter horizons.

This result reaffirms the results discussed above. We see that the regime shift observed in 2020, triggered by COVID-19, has altered the dynamics of return differences between value and growth investment strategies toward a more balanced regime and where return differences now tend to be more easily corrected, which could influence investors’ future decisions when choosing between value or growth strategies.

It is worth noting that the methods employed in this study provide inherent robustness. The ARFIMA model captures long-range dependence without requiring explicit modeling of breakpoints, while the Markov-switching dynamic regression (MS-DR) model detects structural changes endogenously through changes in regime probabilities. These features allow the analysis to remain stable and reliable even in the presence of unobserved or gradual structural shifts. This methodological design reinforces the validity of our findings without the need to impose arbitrary breakpoints or alternative variable definitions.

## 5. Concluding remarks

This study has explored the dynamic behavior of the return difference between value and growth investment strategies, with particular emphasis on the structural changes caused by the COVID-19 pandemic. By employing fractional integration and Markov switching dynamic regression (MS-DR) models, we have provided a comprehensive and robust analysis of the persistence and regime shifts in the relationship between these two strategies over time.

The results show that, prior to the pandemic, the difference between value and growth investment strategies remained in a state where deviations between the two were not easily corrected. This means that when there was a return difference between the two strategies; this return difference persisted over time, with no clear tendency to return to equilibrium or to a more neutral position (reversion to the mean). The changes were maintained over long periods without rapid self-correction. After March 2020, coinciding with the pandemic, the system moved into a new regime of moderate persistence with a tendency to revert toward the mean (equilibrium), with a value of d = 0.99. This reflects an important structural change in the dynamics of the disequilibrium between value and growth strategies. Now, the return differences show a greater tendency to correct themselves and return to equilibrium over time, in contrast to the previous behavior in which the differences persisted longer without reversing.

The Markov switching dynamic regression model identified two distinct regimes in the dynamics of the return difference between value and growth strategies. Regime 0, which was characterized by high persistence and no reversal, dominated the period from January 2009 to March 2020, with a 99.966% probability that the series would remain in this regime. After the pandemic, Regime 1 became predominant, reflecting moderate persistence with a trend toward reversion to the mean, with a 99.9% probability of remaining in this regime. The transition probabilities confirm that, once the system enters Regime 1, it is unlikely to revert to Regime 0, indicating a permanent structural change in the dynamics of disequilibrium.

The evidence of this structural shift has important implications for investors and asset managers. The evolving behavior of the value–growth return differential suggests that strategies relying on pre-pandemic dynamics may no longer be optimal. Instead, a more adaptive framework, one that incorporates changing persistence and regime-switching behavior, may provide more accurate insights into long-term performance and relative strategy valuation.

In sum, this study contributes to the literature by empirically documenting a regime-dependent structural change in the persistence of the value–growth return spread. Our approach demonstrates the utility of fractional integration and regime-switching models in capturing the complex temporal dynamics of financial markets. These results not only deepen our understanding of style investing in the context of structural breaks but also highlight the need for flexible, time-sensitive investment strategies in response to evolving market conditions.
